# Have tsetse flies disappeared from Brazzaville town?

**Published:** 2009-08-28

**Authors:** Lisette Kohagne Tongué, Philemon Mansinsa Diabakana, Patrick Bitsindou, Francis Jacques Louis

**Affiliations:** 1 Programme sous régional de lutte contre la trypanosomiase humaine africaine, Organisation de Coordination pour la lutte contre les Endémies en Afrique Centrale (OCEAC).; 2 Programme national de lutte contre la trypanosomiase humaine africaine de la République Démocratique du Congo.; 3 Direction de la lutte contre la maladie, Ministère de la Santé, des Affaires Sociales et de la Famille du Congo.

**Keywords:** Disappearance, Glossina, sleeping sickness, Congo

## Abstract

**Background:**

From 1980 to 1985, the zoological park of Brazzaville was the only tsetse resting site located in downtown which supplied others temporary sites. The last trapping survey carried out in this area in 1987 showed that there were no more tsetse flies. Knowing that areas free of tsetse used to be reinvaded many years later, we have carried out an entomological survey in the area with the aim to verify what has happened more than twenty years later; given that suitable environmental conditions for Glossina are still available.

**Methods:**

Sixteen pyramidal traps were set out at the edge of the forest, along paths and around animal’s cages and were examined twice a day, at 10 a.m. and 4 p.m. during four days.

**Results:**

No tsetse fly was captured. Using the formula previously described; the probability of capturing a tsetse fly is 0.002.

**Conclusion:**

The zoological park seems close to be free of tsetse flies. Long-lasting surveys within the town and around are required before stating a complete disappearance of tsetse in the town.

## Background

The Congolese (Republic of Congo) capital, Brazzaville is an old sleeping sickness or Human African Trypanosomiasis (HAT) focus. From 1906 to 1908, all HAT cases diagnosed in the hinterland were brought to Brazzaville for treatment [1]. This operation has favored the endemicity of the disease in the area, thanks to tsetse flies present in many resting sites. Despite many control measures developed in the entire country and particularly in Brazzaville, the disease was still endemic in the early sixties, with eight HAT cases diagnosed in 1963 [2].

Brazzaville lays in the southern limit of the distribution area of *Glossina fuscipes quanzensis* Congo. Many tsetse habitats were described, particularly in ravines and at stream sides such as M’Foa, Tsiéma, Makélékélé, M’Filou and Djoué [3]. Over time, urbanization contributed to the destruction of vegetation which previously provided suitable habitat for tsetse flies. The density of Glossina decreased in many areas, even in the zoological park where the apparent density per trap (ADT) was 0.5 Glossina per trap and per day in 1972 [4]. This area was suitable for Glossina because primary vegetation had been conserved and many vertebrates were tamed there.

In 1989, nineteen HAT cases were passively detected downtown Brazzaville but there it was difficult to establish a local contamination [5]. Meanwhile, the presence of *T. b. gambiense* was confirmed. Some authors suggested that two cycles of sleeping sickness were occurring in the city: first, a predominant human-to-human cycle of trypanosomes with a low degree of virulence; and second, a minor cycle involving an animal reservoir [6]. At the same time, the disease was hyperendemic in areas located on both sides of Brazzaville: Niari focus in the south and Couloir focus in the north. Up to now, many HAT cases are regularly diagnosed in these foci [7]. The persistence of the disease in Niari and Couloir foci, despite the implementation of control measures, increases the fear of an outbreak of HAT in downtown Brazzaville. HAT cases coming from active foci located in the hinterland are regularly diagnosed in Brazzaville. The transmission of the disease could be possible in the centre of the town if tsetse flies are present. In 1986, some authors noticed that there were no more tsetse flies in Brazzaville town [8].

What is the situation more than twenty years later? In order to respond to this question, we have carried out an entomological survey in the zoological park of Brazzaville. This area was chosen because from 1980 to 1985, it was the only Glossina resting site which supplied temporary sites to the town [8].

## Methods

### Study area

**Figure 1:**
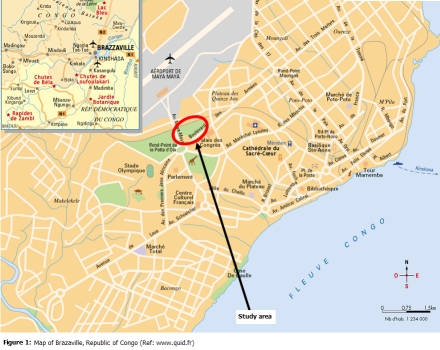
Map of the Brazzaville town, Republic of Congo (Ref. www.quid.fr)

Created in 1952, the zoological park of Brazzaville was the greater park of “Afrique Equatoriale Française (AEF)”. It is located in the centre town of Brazzaville ( Figure 1 ), a part of the wildlife reserve of “Patte d’oie” and is subdivided into three parts: the wild forest, the safari park and a play area. It is a rainy forest zone, conserved in its natural condition. Before the civil war of 1997, this zoo harbored many animals such as primates, reptiles (crocodiles, big lizards, etc.) bovid and suids. Now, only some monkeys, tortoises, snakes and rabbit stew are present.

### Methodology

**Figure 2:**
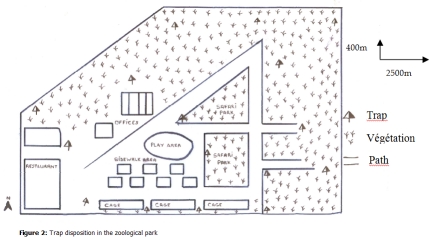
Trap disposition in the zoological park

Sixteen pyramidal traps [9] with a permanent collecting device were used. Traps were set out at the edge of the forest, along paths and around animal’s cages ( Figure 2 ). Each trap site was cleared sufficiently to ensure reasonable visibility of the trap and was examined twice a day, at 10 a.m. and 4 p.m. during four days.

## Results

No tsetse fly was captured. Using the formula previously described [10]; the probability of capturing a tsetse fly is 0.002.

## Discussion

This study was carried out during the rainy season (October) when environmental conditions are appropriate for tsetse flies. The low probability of presence of tsetse in suitable conditions in this previous resting site allows us to state that tsetse flies are rare in the area. We didn’t set traps inside the wildlife reserve. But traps set out around our study area could have caught tsetse flies if there were present in the reserve. The absence of tsetse flies in this area previously known as an overfull area of Glossina could be supported by several reasons among which the trapping conducted in the area between 1980 and 1985 [11, 12]. It was noticed in 1980 that, the ADT which was 0.5 in 1972 rose to 14.5 Glossina per trap and per day [13]. Re-infestation easily operated because Glossina were present in Mbamou Island, situated in the Stanley Pool, upstream from Brazzaville. The area was then appropriate for studies on vector control improvement. That’s how after five years of trapping, a survey conducted in 1986 reported a total disappearance of tsetse flies population [8]. More than twenty years later, the present survey confirms this observation. A re-infestation didn’t occur despite the unceasing presence of Glossina in Mbamou Island. In our knowledge, no study has described an eradication of tsetse flies in this infested area known to be overflowing of tsetse.

In 1992, a progressive replacement of *G. fuscipes quanzensis* by *Glossina palpalis palpalis* in the neighborhood was reported [14]. These species have a great capacity of ecological adaptation. They are both opportunists and ubiquitous as for their habitats as well as their hosts. It seemed thus obvious that at least one of them would have re-infested the zoological park as it has occurred between 1977 and 1980. In addition, these species have a great capacity for dispersal that facilitates their reintroduction in areas previously been under control. One could therefore argue that, re-infestation did not occur, due to the human population growth. In fact, the population of Brazzaville which was 128 000 inhabitants in 1960 increased to 1 247 857 in 2002 [15].

It is known that high population growth could eliminate tsetse flies from large areas, irrespective of control activities [16]; this has been reported in the urban focus of Kinshasa, facing Brazzaville, where some quarters are free of tsetse because of the population growth [17]. The assumption is that human population density is correlated with the area of tsetse habitat cleared for cultivation or housing estate [18]. From 1985 to 2002, the number of inhabitants was multiplied by 8 and the urbanised area by 6.7 [15]. It was already pointed out that a vector control activity was progressively achieving itself in downtown Brazzaville, due to urbanisation and the implementation of market gardening along streams and ravines [8]. One could suppose that tsetse flies coming from infested areas are stopped by unsuitable microclimate existing in the vicinity of the zoo. seeing that Brazzaville has exceeded its natural boundaries materialized by streams “Tsiémé” to the north and “Mfilou” to the west and the periphery of the town is now very far from the centre where the zoological park is located. Besides, in the end of 1990s, a civil war upset Brazzaville quietness. Animals living in the zoological park were either killed, or released. The absence of blood meals for tsetse may have compromised their survival in the zoological park. Since the absence of Glossina in the neighborhood of Brazzaville such as Mbamou Island is not yet reported, one could suppose that the periphery of the town may be infested by Glossina and still at risk of HAT.

## Conclusion

We can’t assume that tsetse flies have completely disappeared in Brazzaville town only in the regards of our results. Nevertheless, this result suggests that the zoological park is close to be free of tsetse. A long-lasting survey is needed within the town and around in all potential areas before stating a complete disappearance of tsetse flies in the town. The complete disappearance of Glossina in this area would contribute to the elimination of HAT.
